# A Simple and Sensitive Approach for Ochratoxin A Detection Using a Label-Free Fluorescent Aptasensor

**DOI:** 10.1371/journal.pone.0085968

**Published:** 2014-01-21

**Authors:** Zhenzhen Lv, Ailiang Chen, Jinchuan Liu, Zheng Guan, Yu Zhou, Siyuan Xu, Shuming Yang, Cheng Li

**Affiliations:** 1 College of Food Science, Sichuan Agricultural University, Ya'an, China; 2 Institute of Quality Standards & Testing Technology for Agro-Products, Key Laboratory of Agro-product Quality and Safety, Chinese Academy of Agricultural Sciences, Beijing, China; 3 Key Laboratory of Agri-Food Quality and Safety, Ministry of Agriculture, Beijing, China; University of Helsinki, Finland

## Abstract

Ochratoxin A(OTA) is found to be one of the predominant contaminating mycotoxins in a wide variety of food commodities. To avoid the risk of OTA consumption, the detection and quantitation of OTA level are of great significance. Based on the fact that ssDNA aptamer has the ability to form a double-strand structure with its complementary sequence, a simple and rapid aptamer-based label-free approach for highly sensitive and selective fluorescence detection of OTA was developed by using ultra-sensitive double-strand DNA specific dyes PicoGreen. The results showed that as low as 1 ng/mL of OTA could be detected with a dynamic range of more than 5 orders of magnitude which satisfies the requirements for OTA maximum residue limit in various food regulated by European Commission. With the specificity of aptamer, the assay exhibited high selectivity for OTA against two other analogues (N-acetyl-l-phenylalanine and zearalenone). We also tested the aptasensor practicability using real sample of 1% beer spiked with a series of concentration of OTA and the results show good tolerance to matrix effect. All detections could be achieved in less than 30 min, which provides a simple, quick and sensitive detection method for OTA screening in food safety and could be easily extend to other small molecular chemical compounds detection which aptamer has been selected.

## Introduction

Ochratoxin A (OTA), a type of mycotoxin, is a secondary metabolite derived from *Aspergillus* and *Penicillium* strains[1]. The toxin could contaminate a large variety of commodities, such as nuts, coffee, cereals, beer, and wine[2]. Some researchers also indicated that OTA could exist in soil ecosystem[3] and indoor environments[4], e.g., damp homes and buildings. As the most toxic and prevalent toxin of the ochratoxins group, OTA has been shown to be weakly mutagenic and can cause immunosuppression as well as immunotoxicity[5], [6] and considered to be a potential carcinogen(group 2B) by the International Agency for Research on Cancer(IARC). Therefore, the rapid and sensitive sensing platforms for OTA detection is highly required in food safety and environmental monitoring.

Analysis of OTA is usually performed by conventional analytical techniques, including thin layer chromatography (TLC)[7], [8] and high-performance liquid chromatography (HPLC)[9]–[11], which are widely accepted for their low detection limit. However, the approaches based on these techniques involve time-consuming sample preparation steps and require sophisticated equipments and trained personnel, which could not meet the requirements of on-site and rapid detections. Immunoassays are based on the ability of a specific antibody to distinguish the three-dimensional structure of OTA and various of immunological methods have been developed for OTA detection, including enzyme linked immunosorbent assays (ELISA)[12]–[14], surface plasmon resonance (SPR)[15], fluorescence polarization immunoassay (FPIA)[16], enzyme-linked electrochemical immunosensor[17], [18], array immunosensors[19]. Although these methods present good sensitivity and selectivity with the potential for high-throughput screening, they often suffer from the possibility of false positive and negative screening results and matrix effect. Note that all of these immunosensors are heavily reliant on the quality of the antibodies and the preparation of antibody is laborious, expensive, time-consuming and may be susceptible to problems with stability or modification.

To overcome the above limitations, aptamers appear as new promising recognition molecules for analytical applications[20]. Aptamers are selected in vitro by systematic evolution of ligands by exponential enrichment (SELEX), and are single-stranded DNA or RNA molecules that can bind various target ligands including small-molecule drugs, peptides, proteins and cells with high affinity and specificity[21], [22]. Besides their high target binding capability, aptamers show several overwhelming advantages over traditional protein antibodies, such as target diversity, high stability, easy of synthesis and modification for applications[23], [24]. The aptamer selected for OTA not only exhibits a dissociation constant in the nanomolar range but does not bind with compounds whose structures are similar to OTA [2]. A variety of analytical techniques based on this aptamer have been developed, including colorimetric assay[25], [26], fluorescence assay[27]–[29], electrochemical aptasensor[30]–[32] and aptamer-based test strip format has also been proposed for OTA determination[33]. Among these detection methods, the analysis based upon fluorescence has the advantages of simplicity, rapidity, less expensive and more suitable for automation, however, these fluorescence assays require fluorophore-labeled aptamer. Such step would not only make experiments relatively more expensive and complex, but may also affect the binding affinity between the OTA and aptamer and influence the sensitivity for detection[34]. Therefore, new fluorescence analysis, especially those rapid, simple, sensitive and cost-effective methods are highly desired for quantitative OTA detection in food safety.

Herein, we reported on the development of a facile aptamer-based label-free approach for highly sensitive and selective fluorescence detection of OTA using commercially available PicoGreen(PG) to transduce the fluorescent signal of the double strand DNA duplex formed between remaining aptamer and its complementary strand. The assay shows high sensitivity and offers great potential for on-site and high-throughput analysis, which is conducive to detect mycotoxin in agricultural products.

## Materials and Methods

### Reagents and chemicals

All the oligonucleotide sequences used in this study were synthesized and purified through HPLC by Sangon Biotechnology Co., Ltd. (Shanghai, China), and sequence information was listed in Table 1. The fluorescent dye PG (200× concentrated) was purchased from Invitrogen (CA, USA). OTA and zearalenone (ZEN) were purchased from Fermentek (Jerusalem, Israel). N-acetyl- l-phenylalanine (NAP) was purchased from J&K Acros Organics (Beijing, China). All other reagents were of analytical grade and bought from AccuStandard (Beijing, China).

**Table 1:** Aptamer and complementary sequences used in this study.

Description	Sequence(5′-3′)
Ochratoxin A aptamer	GATCGGGTGTGGGTGGCGTAAAGGGAGCATCGGACA
Complementary strand	TGTCCGATGCTCCCTTTACGCCACCCACACCCGATC
Chloramphenicol aptamer	ACTTCAGTGAGTTGTCCCACGGTCGGCGAGTCGGTGGTAG
Complementary strand	CTACCACCGACTCGCCGACCGTGGGACAACTCACTGAAGT

### Fluorescent Detection of OTA

For OTA detection by our method, 25 µL of OTA aptamer solution (0.5 µM) was mixed with 50 µL 1× OTA binding buffer (10 mM Tris-HCl, 120 mM NaCl, 5 mM KCl, 20 mM CaCl2, pH 8.5) containing different concentrations of OTA in microplate wells. Samples were incubated at room temperature for 20 min. Then, 25 µL of 0.5 µM complementary strand of OTA aptamer and 10 µL 10× PG was added to the sample wells. After incubation for 3 min, the fluorescence intensities were scanned with LS-55 Fluorescence Spectrometer (Perkin e Elmer, Norwalk, CT) with an excitation of 480 nm and emission of 523 nm or recorded using a multifunction microplate reader (Tecan Infinite 200, Tecan Austria GmbH, Austria) with an excitation of 480 nm and emission of 520 nm. Each value is the mean of triplicates.

## Results and discussion

### Principle of the label-free fluorescent aptasensor

**Figure 1:**
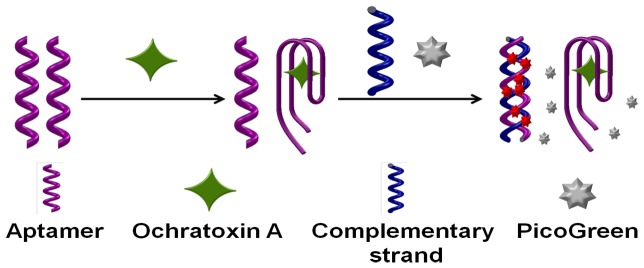
Schematic illustration of fluorescent detection of OTA by a label-free aptasensor.

PicoGreen reagent is an asymmetric cyanine dye which does not fluoresce when free, but upon binding to dsDNA it exhibits a >1000-fold fluorescence enhancement while no significant fluorescence change can be observed when it binds to ssDNA[35]. Using the commercially available PicoGreen dye as the signal probe as low as 25 pg/mL dsDNA can be detected. Based on PG's ultra-selectivity for trace dsDNA and dramatic increase in fluorescent emission upon interact with dsDNA, the label-free aptasensor was developed herein. The sensing mechanism of the proposed method for OTA detection is illustrated in Figure 1. In the presence of OTA, the conformation of OTA's aptamers changes from random coil structure to G-quardruplex structure [25]. After addition of the complementary strands and PG, the complementary strands will hybrid with the remaining free aptamers which didn't bind with the targets. With the formation of the double strand DNA, the PG inserts into the minor groove of dsDNA duplex and results in the significantly fluorescent enhancement. Therefore, quantitative analysis of OTA can be achieved by monitoring the fluorescence intensity change of PG, from where the quantity of OTA is inversely proportional to the fluorescence intensity.

### Optimization of the aptasensor reaction time

**Figure 2:**
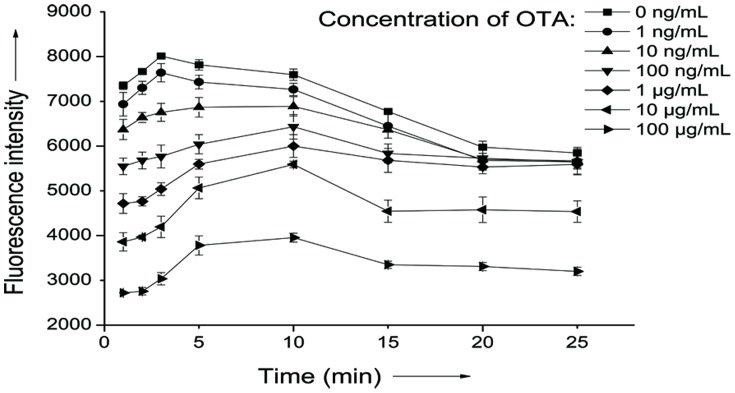
Optimization of the incubation time after aptamer complementary strand and PicoGreen were added into the different concentration of OTA and aptamer mixture.

Since our approach relies on the fluorescence intensity change of PG, it is important to ensure that the decrease of fluorescence is attributed only to the specific interaction between the OTA and its aptamer. So the intercalation time between PG and dsDNA duplexes and also aptamer and complementary strand hybridization time were optimized before performing OTA assay. As shown in Figure 2, for low concentrations of OTA, the fluorescence intensity peaked at 3 min and then decreased gradually due to the fluorescence decay of PG. For high concentrations of OTA, the fluorescence intensity reached a maximum at 10 min in which less free aptamers are available to form dsDNA for PG interaction. The results may suggest that there is a competition for aptamer between aptamer/OTA complex and aptamer/complemantary strand DNA duplex from 3 min to 10 min and the binding affinity of aptamer/OTA complex was stronger than that of the DNA duplex which leads to the increase of fluorescence. However, as a whole, an about 3 min of dsDNA formation as well as PG interaction time was selected as it give a maximum fluorescence intensity difference for various concentrations of OTA which means high sensitivity.

### Detection of OTA with the label-free fluorescent aptasensor

**Figure 3:**
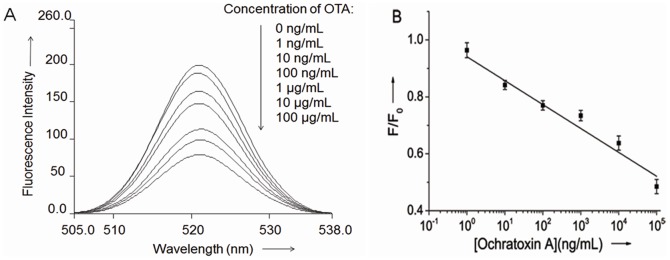
Label-free aptasensor using PG for detection of OTA. (A) Fluorescence spectra of the PG/aptamer duplex mixture in the presence of various concentrations of OTA. (B) Calibration plot relative F/F_0_ of the PG/aptamer duplex mixture against different concentrations of OTA. F_0_ and F stand for the fluorescent intensity in the absence and presence of OTA.

As shown in Figure. 3a, the presence of increasing concentration of OTA from 1 ng/mL to 100 µg/mL leads to a gradual decrease of the fluorescence intensity of the intercalated PG. The linear relationship of the fluorescence signal decrease (F/F_0_) vs the OTA concentration in the Figure 3b indicated that OTA concentration can be quantitatively derived, and as low as 1 ng/mL OTA can be detected with a dynamic range of more than 5 orders of magnitude. The aptasensor exhibits higher sensitivity than other OTA sensors which utilize fluorescent[27], [36] (9.64 µg/L; 7.48 µg/L) and colorimetric[25] (8.06 µg/L)detection methods. Such high sensitivity may be attribute to the first formation of aptamer/OTA complex which avoid the competition between the aptamer/OTA complex and DNA duplex according to Yan[37]. To illustrate this, we also performed the assay with same condition except that the aptamer was firstly mixed with complementary strand and PG before the OTA addition. The switch from DNA duplex to aptamer/OTA complex truly gives a low detection limit of about 100 ng/mL (data not shown).

### Specificity of the label-free fluorescent aptasensor for OTA

**Figure 4:**
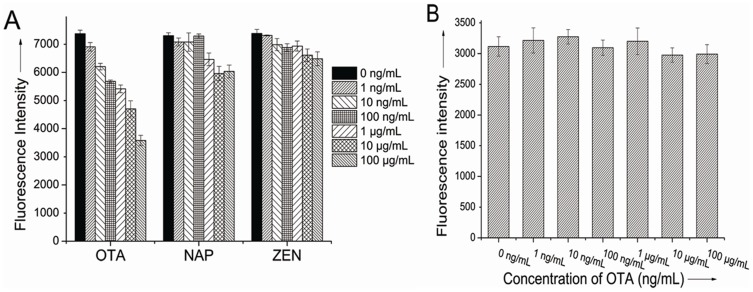
Selectivity evaluation the aptasensor for OTA. (A) Against NAP and ZEN in same series concentrations. (B) Using a chloramphenicol aptamer and its complementary strand.

The specificity of the assay protocol relies on the high selectivity of the aptamer. In order to determine the specificity of this method, we tested the sensing platform against two non-specific targets: N-acetyl- l-phenylalanine (NAP) and zearalenone (ZEN). As shown in Figure 4a, the presence of ZEN had a negligible effect on the detection of OTA and there only about 8.8% decrease of fluorescence intensity at the high concentration of 100 µg/mL while the addition of NAP at 100 µg/mL resulted in an about 15% decrease of fluorescence intensity. NAP molecular structure represents one part of OTA [27], which to some extent, still possesses the combination ability with anti-OTA aptamer. However, the ZEN is distinctly different from OTA, so the fluorescence responses are almost negligible.

More control experiments using chloramphenicol's aptamer[38] and its complementary strand (Table 1) were performed under the condition similar to OTA's aptamer. The results showed that there was no obvious decrease in the fluorescence intensity at various concentrations of OTA (Figure 4b). These results clearly indicated that the aptamer-based fluorescence aptasensor is highly specific for OTA determination.

### Practicability of the label-free fluorescent aptasensor for OTA

**Figure 5:**
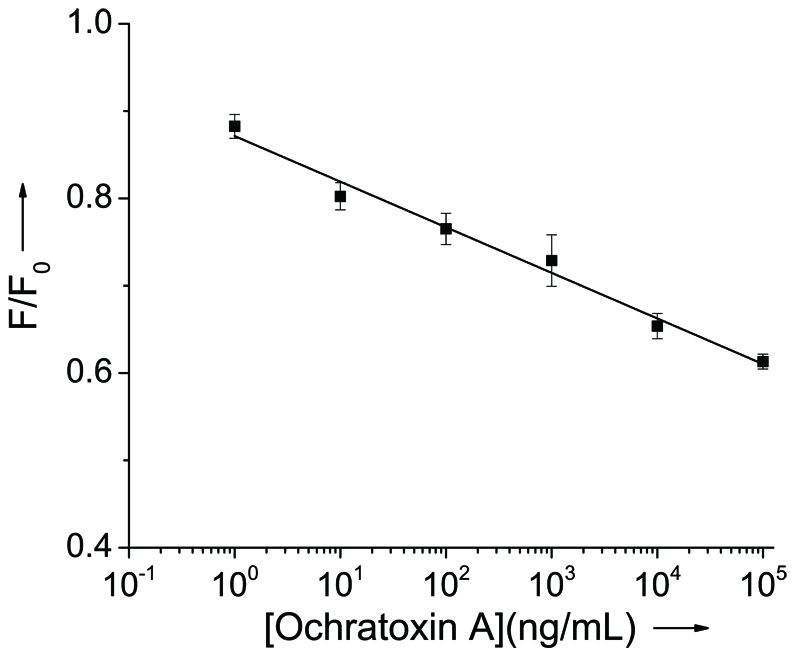
Calibration plot relative F/F_0_ of the PG/aptamer duplex mixture against different concentrations of OTA in 1% beer. F_0_ and F stand for the fluorescent intensity in the absence and presence of OTA.

In order to evaluate the feasibility and reliability of the proposed sensing system for practical applications, detection of OTA in the beer sample was challenged by our system. Different concentrations of standard solutions of OTA were added into the 1% beer and detection process was the same as in the buffer solution. As shown in Figure 5, concentration of OTA down to 1 ng/mL was detected and the linear range was wide from 1 ng/mL to 100 µg/mL. This result suggested that the sensor can be applied to the analysis of OTA in real food samples.

## Conclusions

In summary, a simple and rapid aptamer-based label-free detection for highly selective and sensitive fluorescence detection of OTA had been developed. The high sensitivity of the developed sensing system mainly attributes to the ultra-sensitivity of the PG for trace dsDNA while the high selectivity is mainly due to the aptamer exhibits a dissociation constant in the nanomolar range to OTA. The linear dynamic range and its detection sensitivity were found to be from 1 ng/mL to more than 100 µg/mL and 1 ng/mL of OTA respectively. Furthermore, the entire detection could be completed in less than 30 min. Due to its simple design, easy operation, fast response and high sensitivity and selectivity, the proposed biosensor may provide significant improvements in OTA screening for food safety monitoring. Importantly, it is a nearly universal method due to the exceptional dsDNA selectivity of PicoGreen and the principle can be extended to the detection of other targets which aptamers have been selected.
